# Grade 3 well-differentiated neuroendocrine tumor of the rectum: a case report

**DOI:** 10.1186/s40792-020-00893-y

**Published:** 2020-06-12

**Authors:** Misato Ito, Yasumitsu Hirano, Toshimasa Isii, Hiroka Kondo, Liming Wang, Masahiro Asari, Nao Obara, Shigeki Yamaguchi

**Affiliations:** Department of Gastroenterological Surgery, Saitama Medical University International Medical Center, 1397-1, Yamane, Hidaka-City, Saitama-Pref Japan

**Keywords:** Neuroendocrine tumor G3, Colon, Rectum

## Abstract

**Background:**

The 2019 revised World Health Organization (WHO) classification of tumors of endocrine organs classifies grade 3 gastroenteropancreatic neuroendocrine neoplasms (GEP-NEN G3) into well-differentiated tumors (NET G3) and poorly differentiated carcinomas (NEC G3). There are few reported cases of NET G3 occurring in the rectum.

**Case presentation:**

A 71-year-old man complained of bright red blood in his stool. Total colonoscopy revealed a mass in the lower rectum. Pathologic examination yielded a diagnosis of group 1. Computed tomography revealed swollen paraintestinal lymph nodes and multiple liver metastases. We performed laparoscopic abdominoperineal resection not only to avoid the unbearable symptoms caused by tumor growth but to make a pathological diagnosis. The tumor measured 3.5 × 2.8 cm, and the pathological diagnosis was stage IV neuroendocrine carcinoma. He underwent chemotherapy with irinotecan plus cisplatin, followed by carboplatin plus etopside, but his disease did not respond to either regimen. Twenty-seven months after surgery, he died of his disease. Upon re-examination of the surgical specimen, the tumor was consistent with the 2019 WHO classification of NET G3.6

**Conclusion:**

A definite diagnosis of NET G3 or NEC G3 must be made to determine the appropriate treatment strategy for patients with GEP-NEN G3. Further case reports and case series are needed to establish the optimal therapy.

## Introduction

The 2019 revised World Health Organization (WHO) classification of tumors of endocrine organs classifies grade 3 gastroenteropancreatic neuroendocrine neoplasm (GEP-NENs) into well-differentiated neuroendocrine tumors (NET G3) and poorly differentiated neuroendocrine carcinomas (NEC G3). The concept of NET G3 was first described in the WHO 2017 classification of pancreatic tumors related to multiple endocrine neoplasia (MEN) syndromes. There are few reported cases of NET G3 occurring in the rectum, and there are currently no data on antitumor therapy for patients with metastatic colorectal NET G3.

## Case report

A 71-year-old Japanese man had a known colonic polyp, but he did not have it rechecked for 7 years until he began to experience hematochezia. Colonoscopy revealed the tumor of the rectum, and he was referred to our hospital. He had no significant medical history. His father had suffered from lung cancer and a testicular tumor; however, there was no evidence of MEN in his family history. He did not smoke or drink alcohol, and he had no allergies.

**Fig. 1:**
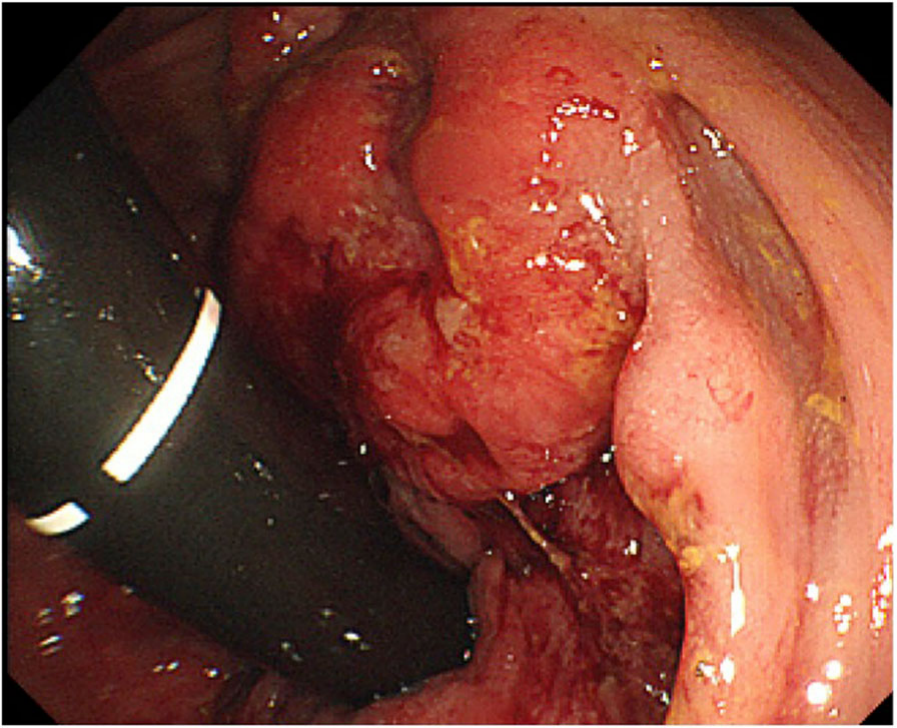
Colonoscopy image showing a mass in the lower rectum, describing a quarter circle and in contact with the anal canal

**Fig. 2:**
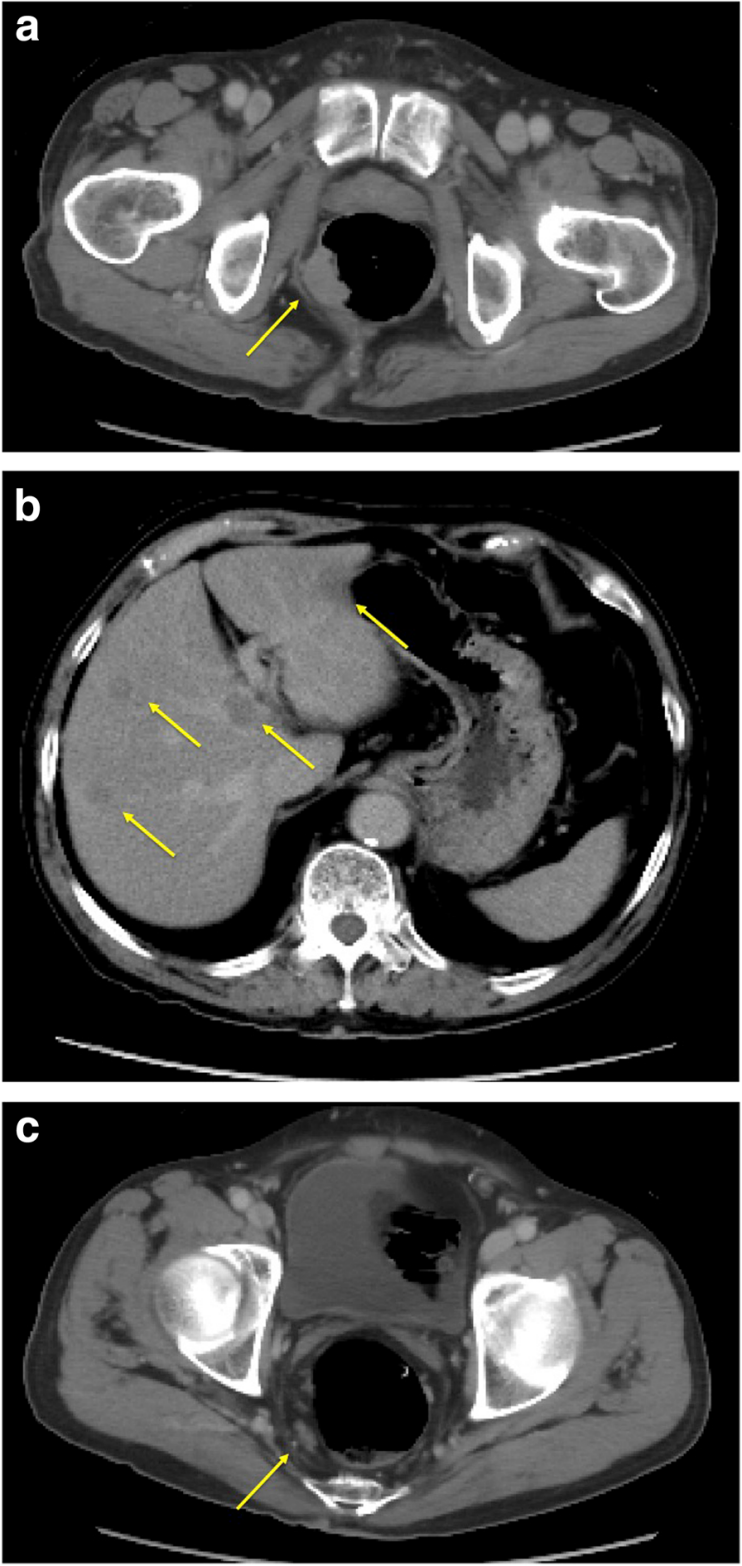
Computed tomography (colonography) image. **a** This reveals a tumor in the lower rectum with suspected invasion of the right levator ani muscle. **b**, **c** The paraintestinal lymph nodes are swollen, and there are multiple liver metastases

**Fig. 3:**
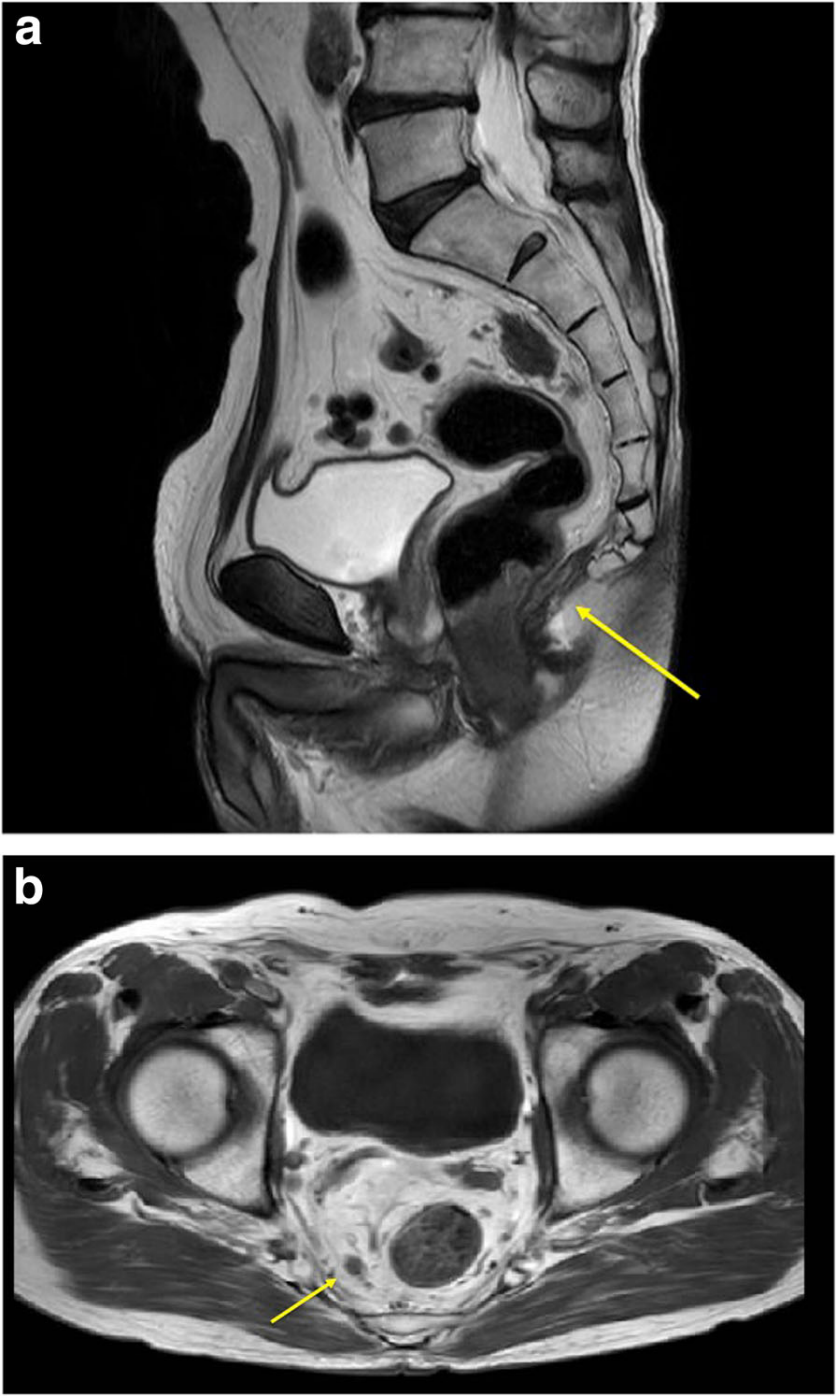
Magnetic resonance imaging. **a** This reveals suspected tumor invasion of the right levator ani muscle. **b** The right lateral lymph nodes were swollen

A mass was palpable on rectal examination, forming a semicircle at the posterior wall of the rectum. Laboratory analysis revealed a total peripheral leukocyte count of 7100/mm^3^, with normal tumor markers (carcinoembryonic antigen [CEA], 2.3 ng/mL; cancer antigen 19-9 [CA 19-9], 19.6 U/mL). His height was 165.5 cm, and his weight was 57.9 kg, giving a body mass index of 21.14 kg/m^2^. Total colonoscopy revealed a mass in the lower rectum (Fig. 1). The tumor described a quarter circle around the rectum and was in contact with the anal canal. Pathologic examination of a biopsy specimen yielded a diagnosis of group 1 (ulcerative lesion with generated epithelium). Computed tomography (colonography) revealed a tumor in the lower rectum, suspicious for invasion of the right levator ani muscle, swollen paraintestinal lymph nodes, and multiple liver metastases (Fig. 2). We judged that the liver metastases were unresectable because they occurred in both lobes with adjacent to major hepatic vein. There were no metastases to the lungs or other structures. Magnetic resonance imaging confirmed the presence of swollen right lateral lymph nodes (Fig. 3).

**Fig. 4:**
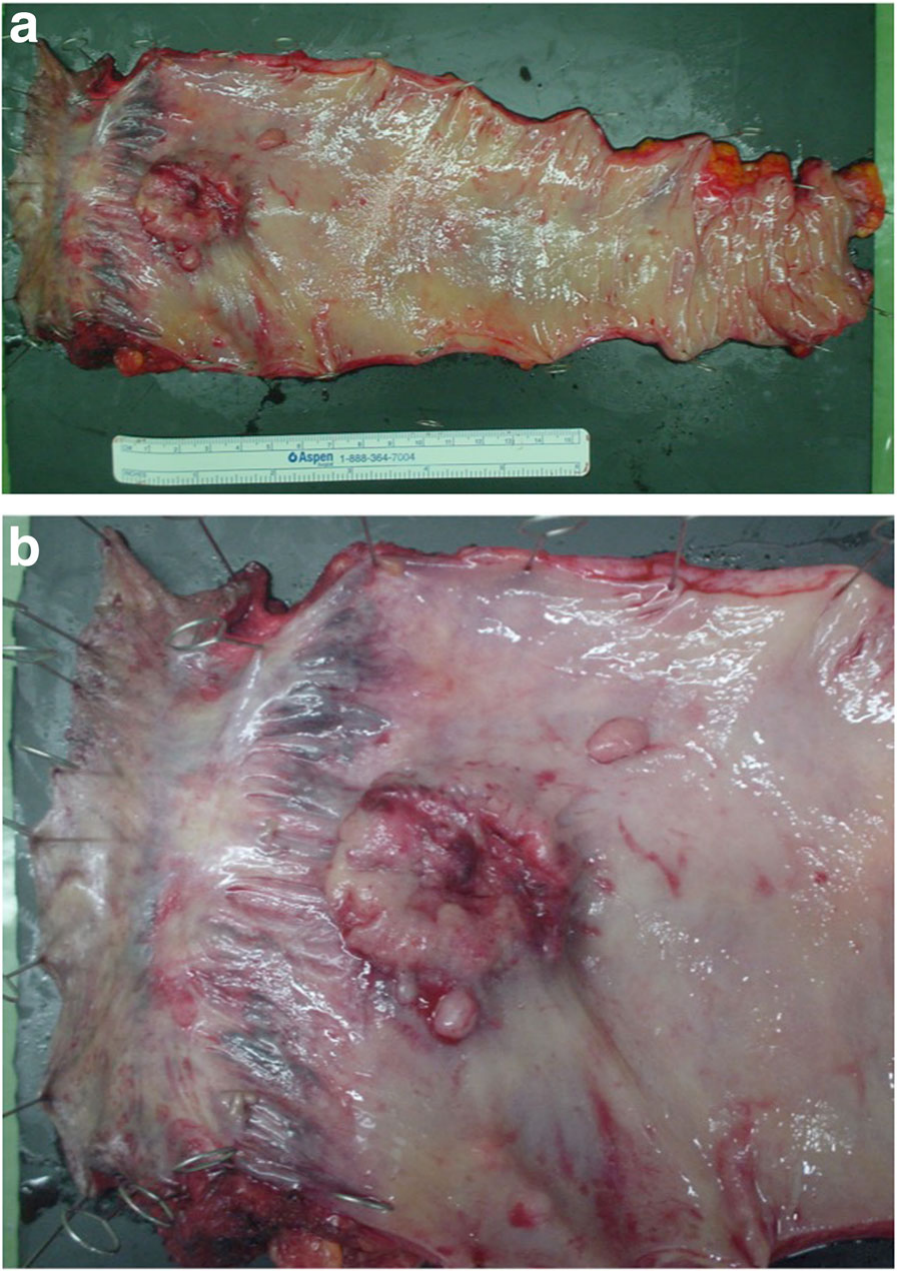
Specimen: **a**, **b** The tumor in the wall of the rectum measures 3.5 × 2.8 cm

We performed laparoscopic abdominoperineal resection of the rectum and lymph node dissection, with resection of the right pelvic plexus not only to avoid the unbearable symptoms caused by tumor growth but to make a pathological diagnosis of the rectal tumor. The operative time was 279 min, and the intraoperative blood loss was 200 mL. The tumor was located in the lower wall of the rectum and measured 3.5 × 2.8 cm (Fig. 4).

**Fig. 5:**
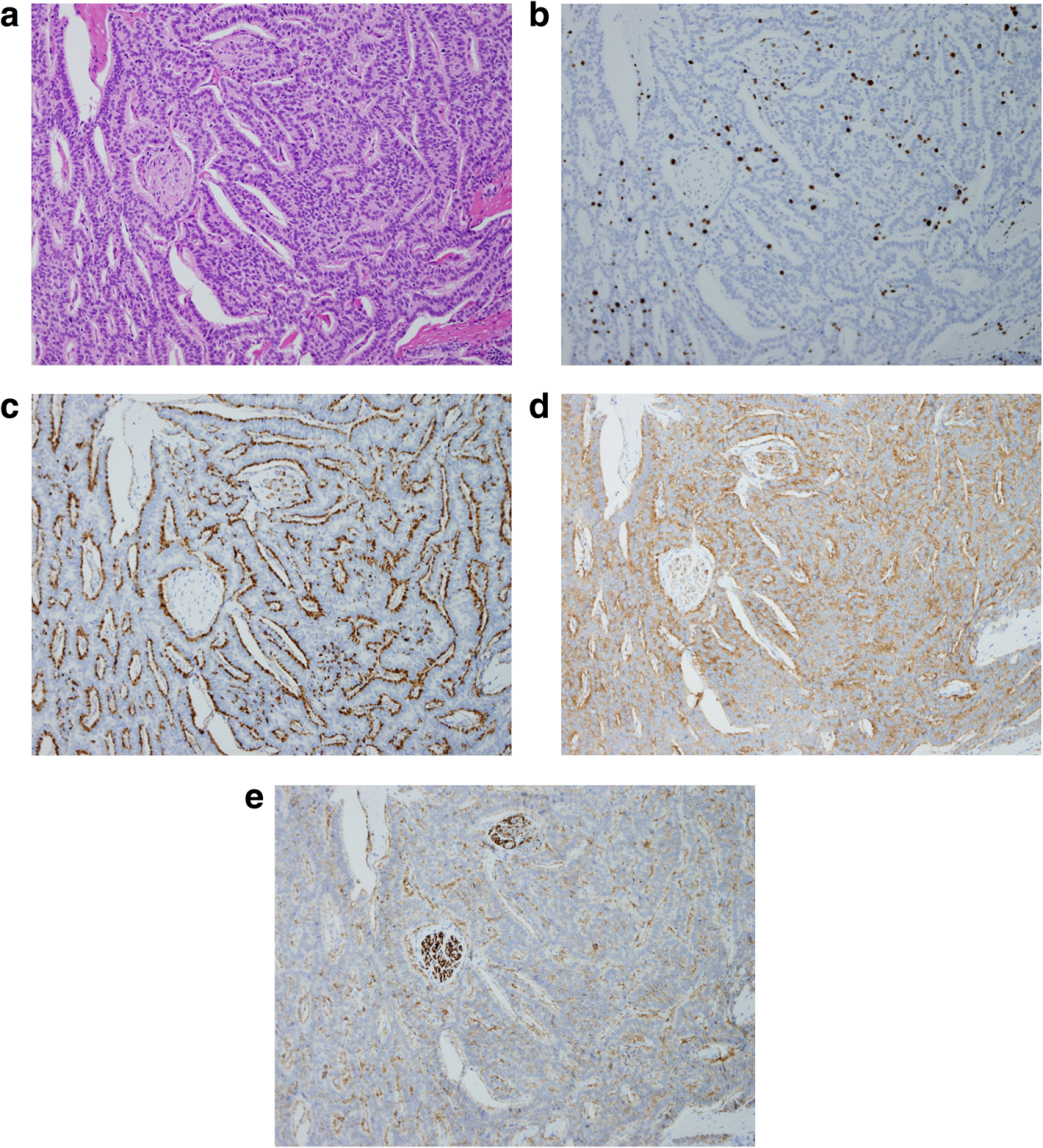
Histopathologic findings. **a** Chromatin is increased in the atypical cells, which feature mitotic figures. Invasion involves the subserosa. Hematoxylin-eosin staining (magnification × 20). **b** MIB-1 index > 20%. **c** Positive staining for chromogranin A. **d** Positive staining for synaptophysin. **e** Positive staining for CD56

Histological examination showed the rectal tumor with subserosal invasion (pT3) involving venous and lymphatic invasion. Immunohistochemical staining revealed that diffuse staining for synaptophysin and chromogranin A, and focal staining for CD56, and MIB-1 index was over 20%. The results of CK7, CK20, and S-100 protein were negative. The patient’s Ki67 index was 25 percent. The pathologic diagnosis of the day was neuroendocrine carcinoma, stage T3N3M1(H2), according to the 2010 WHO classification of tumors of endocrine organs (Fig. 5). The patient was discharged on the tenth postoperative day.

Two months after surgery, the patient began chemotherapy with irinotecan (60 mg/m^2^) plus cisplatin (60 mg/m^2^). In the second and third courses, each dose was reduced by 50% from the standard because of a renal dysfunction. After 3 courses, his liver metastases had increased in size, so we changed the regimen to carboplatin (400 mg/m^2^) plus etoposide (100 mg/m^2^). In the second course, each dose of carboplatin was reduced by 50% from the standard because of a febrile neutropenia. After 2 courses, the liver lesions continued to enlarge. At that point, we discontinued chemotherapy and provided supportive care. Twenty-seven months after surgery, he died of his disease.

Pathologic re-examination of the surgical specimen after he died revealed that the tumor was consistent with the 2019 WHO classification of NET G3.

## Discussion

The incidence of neuroendocrine tumor was 1.09 per 100,000 persons in 1973, increasing to 6.98 per 100,000 persons by 2012 in the USA [1]. The 2019 revised WHO classification of tumors of endocrine organs classifies grade 3 GEP-NENs into NET G3 and NEC G3 categories, indicating well- or poorly differentiated neoplasms, respectively. Cytologically, neuroendocrine tumors usually have abundant granular cytoplasm, which results in a low nuclear to cytoplasmic (N/C) ratio, and stippled chromatin. Cells of NEC G3 neoplasms have a lesser amount of granular cytoplasm and a higher N/C ratio [2, 3]. Immunohistochemically, both NET G3 and NEC G3 have a Ki-67 index of greater than 20% and an amitotic index of greater than 20 per 10 high-power fields. There are some differences in their pathologic characteristics. The rate of positive chromogranin A is 100% in NET G3 and 88.6% in NEC G3, with positive synaptophysin rates of 95.2% and 93.8%, median Ki67-LI values of 28.5% and 80.0%, loss of retinoblastoma protein (Rb) expression in 0% and 54.5%, presence of a KRAS gene mutation in 0% and 48.7%, loss of Rb expression with a KRAS mutation in 0% and 30%, and p53 expression in 0% and 75%, respectively [4, 5]. The expression of Rb and p53 is particularly useful especially in patients who are difficult to differentiate between NET G3 and NEC G3 because the expression rate is high in NEC G3, but neither are expressed in NET G3.

The primary treatment for GEP-NEN G3 is surgery. Tumors greater than 2 cm and those that invade the muscularis propria or with locoregional lymph node involvement should generally be managed similarly to colorectal adenocarcinoma [6]. The high-grade malignancies NET G3 and NEC G3 have already developed distant metastases by the time of the primary resection, and even if the primary lesions are completely removed, they can recur on a long-term basis. For metastatic neuroendocrine carcinoma, the combination of cisplatin and etoposide is recommended as first-line therapy [6]. However, Hijioka et al. reported that NET G3 does not respond to platinum-based chemotherapy [4]. The Ki67 index is significantly higher in neuroendocrine carcinoma; Sorbye et al. and Heetfield et al. reported that patients with Ki-67 less than 55% are less responsive to platinum-based chemotherapy [7, 8]. There are currently no data on antitumor therapy for patients with metastatic colorectal NET G3. The Clinical Practice Guidelines in Oncology: Neuroendocrine Tumors Version 1, 2019, recommends octreotide, lanreotide, everolimus, and peptide receptor radionuclide therapy using 177Lu-dotatate, similar to that used for patients with grade 1 and grade 2 neuroendocrine tumors [9]. There is no known role for systemic adjuvant therapy. In this patient, we diagnosed neuroendocrine carcinoma based on the 2010 WHO classification, and he was treated with platinum-based chemotherapy. We could not change the diagnosis at that time, but we should not select platinum-based chemotherapy because the Ki-67 index of his tumor was 25% that means his tumor was less responsive to this therapy. Although his disease did not respond, he survived 17 months after he discontinued chemotherapy. His treatment course indicates that the tumor growth of NET G3 is slower than that of neuroendocrine carcinoma. Heetfield et al. reported that the median overall survival is 98.7 months for NET G3 and 17 months for neuroendocrine carcinoma [8].

In this case, we treated him as NEC till he died, and the diagnosis was changed due to the revision of the WHO classification in 2019. If he was diagnosed with NET G3 in WHO classification of 2019, he would be treated with the abovementioned therapy that the Clinical Practice Guidelines in Oncology recommended, and his prognosis might be extended [9].

We believe that it is important to distinguish between these 2 classifications to avoid using ineffective treatment modalities, and we should do pathologic re-examination when the disease does not respond for first chemotherapy or discontinued chemotherapy to give the appropriate treatment to NEC patients diagnosed by the WHO 2010 classification.

## Conclusions

A definite diagnosis of NET G3 or NEC G3 is necessary to determine treatment strategy. Study of further case reports and case series should result in discovery of the optimal therapy.

## Data Availability

Data sharing not applicable to this article since datasets were neither generated nor analyzed for the case series.

## Abbreviations

GEP-NEN G3: grade 3 gastroenteropancreatic neuroendocrine neoplasms
NET G3: Well-differentiated tumors
NEC G3: Poorly differentiated carcinomas
WHO: World Health Organization
CEA: Carcinoembryonic antigen
CA 19-9: Cancer antigen 19-9
N/C: Nuclear-to cytoplasmic
EUS-guided FNA: Endoscopic-guided fine-needle aspiration
